# Social norms and webcam use in online meetings

**DOI:** 10.3389/fpsyg.2022.907405

**Published:** 2022-07-26

**Authors:** Sarah Zabel, Genesis Thais Vinan Navas, Siegmar Otto

**Affiliations:** ^1^Institute of Education, Labor and Society, University of Hohenheim, Stuttgart, Germany; ^2^Institute of Psychology, Otto-von-Guericke University, Magdeburg, Germany

**Keywords:** online meetings, webcam use, virtual team, social norms, media richness, videoconferences

## Abstract

Face-to-face meetings are often preferred over other forms of communication because meeting in person provides the “richest” way to communicate. Face-to-face meetings are so rich because many ways of communicating (spoken language and nonverbal cues) are available to support mutual understanding. With the progress of digitization and driven by the need to reduce personal contact during the global pandemic, many face-to-face work meetings have been shifted to videoconferences. With webcams turned on, video calls come closest to the richness of face-to-face meetings. However, webcam use often remains voluntary, and some participants choose not to turn their cameras on. In order to find ways to support webcam use—when desired—we analyzed how social norms in groups affect the decision to activate a webcam in a specific meeting. Based on an online survey with *N* = 333 participants, we found that social norms are related to an individual’s decision to turn on the webcam, even when controlling for group size. If the number of participants with activated webcams in a university meeting increased by 25%, it was 5.92 times more likely that an individual decided to turn their webcam on, too. Furthermore, 81.84% of respondents indicated they would turn on their webcam if participants in a meeting were explicitly asked to do so. The results demonstrate a strong relation between social norms and the decision to activate a webcam in online meetings. They build a basis for enhancing webcam use and enable a greater richness of communication in online meetings.

## Introduction

In recent decades, a globalized economy has necessitated changes in communication channels (Standaert et al., 2021). With many participants distributed across different locations, face-to-face meetings have been replaced by technology-supported meetings (e.g., videoconferences). Beginning in 2020, the COVID-19 pandemic was an additional driver of the digitization of work. In order to minimize personal contact, face-to-face meetings have been replaced by online meetings in a variety of different settings, including schools, universities, and the workplace (Karl et al., 2021). Even after the COVID-19 pandemic passes, it is expected that the majority of business meetings will take place *via* video calls, and virtual meetings will still be a part of many people’s daily lives in the future (Standaert et al., 2021). Therefore, the question arises whether it is beneficial for participants to turn on their webcams. In many cases, seeing each other in meetings more effectively supports the purpose of the meeting, especially when the tasks are ambiguous and misunderstandings can be prevented through the presence of multiple cues (e.g., facial expressions or gestures in addition to speech only; see Lengel and Daft, 1988). Daft and Lengel (1983) referred to the ability of communication channels to facilitate shared meaning as *media richness*. According to Daft et al. (1987), understanding is enhanced through (a) instant feedback, (b) the availability of multiple cues, (c) language variety (whether there is a precise meaning, as there is for numbers, or different meanings that can be conveyed), and (d) personal focus (whether the communication can contain emotions). Besides enhancing understanding, a higher media richness is also likely to increase trust in conversational partners (Babutsidze et al., 2021). Even though videoconferencing will never be as “rich” as in-person meetings, it is the communication channel which comes closest to personal meetings because of the many cues that are available. This leads to several advantages compared to other—less rich—ways of communicating: Turning on the webcam was found to increase creativity and perceived authenticity among the participants (Olson et al., 2012) and participants experience a stronger connection and increased trust (Larsen, 2015). Furthermore, attentiveness and engagement are higher in webcam-supported meetings, which in turn increase meeting effectiveness as soon as participants have become familiar with the technology (Olson et al., 2012). Compared with communication *via* telephone, videoconferences also facilitate discussions and joint decision-making in virtual teams (Hofmann et al., 2013). However, videoconferences with webcams turned on can also reduce team performance, which is likely due to an information overload for participants who see their own video (Hassell and Cotton, 2017). Thus, it has to be weighed up in which contexts the positive outcomes of activated webcams in meetings outweigh potential distractions through seeing one’s video. In many cases, meetings supported by videoconferencing tools are nevertheless popular because they allow participants to maintain direct visual contact with other participants who turn on their webcams and provide further information for interpreting messages.

However, participants are not always willing to switch on their webcams (Bedenlier et al., 2021), and in some cases, such willingness even decreases over time (Castelli and Sarvary, 2021). Studies in the educational context have shown that participants’ main reasons for not turning on their webcams were concerns about their appearance or privacy (see also Bedenlier et al., 2021) or a weak Internet connection (Castelli and Sarvary, 2021). But social norms (descriptive and injunctive) also influence whether someone turns on their webcam (Castelli and Sarvary, 2021). Whereas descriptive norms refer to what is commonly done (i.e., whether other participants turn on their webcams), injunctive norms specify what most people approve of Cialdini and Trost, (1998) and Cialdini et al. (1991). When an individual has to decide for a behavior, they look at what others are doing in the situation. Thus, the descriptive norm provides an information processing advantage for individuals (Cialdini et al., 1991). When entering a meeting where most participants—at least those whose tiles are visible on the newcomer’s screen—have turned on their webcams, the newcomer thereby receives a hint as to what the most effective social behavior might be. In turn, adhering to the injunctive norm (i.e., turning on the webcam because others approve of it) promises social acceptance (Cialdini and Trost, 1998). Researchers have shown that social norms shape individual behavior in a variety of contexts (e.g., Goldstein et al., 2008), for example, downloading an app for the social good (Zabel et al., 2021, submitted manuscript).^1^ Although in previous studies, the most commonly indicated reasons for not activating a webcam were different, we expect social norms to have a large influence on webcam activation in meetings. Because to date, most studies on webcam activation behavior have been conducted in an educational context, we extend previous research by additionally examining meetings in a professional context. In university lectures, where speaker and audience are often distinct, turning on a webcam can be beneficial for other reasons as it is in many professional contexts. In speaker-audience contexts, audience responsiveness is related to less public speaking anxiety on the part of the speaker and a higher quality of speech (MacIntyre et al., 1997). Moreover, the reasons for turning on the webcam or not can also be different depending on the centrality of the communication structure, that is, whether there is one speaker or participants of the meeting are interacting with one another. In the latter case, there might be a higher willingness to turn on the webcam in general, because participants want to engage in the meeting, as compared to speaker-audience contexts (Marlow et al., 2017). Based on these findings on webcam use in online meetings, we define the following research questions:

RQ1: Are descriptive and injunctive social norms related to webcam activation in online meetings?

RQ2: Is this relation (RQ1) different in a professional versus a university context?

RQ3: What are the reasons for not turning on the webcam in both contexts?

## Materials and methods

Data were collected *via ad hoc* sampling with an online questionnaire. Two students recruited the participants between June 10, 2021 and July 6, 2021 as a part of their theses. The aim was to reach German-speaking participants with a minimum age of 16 years who possess a webcam and have already participated in an online meeting. Therefore, the students posted the link to the survey on their social network profiles (Facebook and Instagram) and recruited personal contacts.

### Sample

From 393 participants who met the sample requirements (i.e., indicated that they possess a webcam and had participated in online meetings), seven cases were excluded due to implausible completion speeds. We focused on participants whose last meeting was either in a university context (*n* = 200) or a professional context (*n* = 133). The mean age of the sample was *M* = 22.93 (*SD* = 4.53) for the university group and *M* = 37.83 (*SD* = 12.77) for the professional group. The gender distribution was similar in the two groups—61.0% of the university group and 65.4% of the professional group were women, 1.5% (university) and 0.7% (professional) were inter/diverse, and 4.0% (university) and 7.5% (professional) did not specify their gender identity.

### Measures

Participants were asked whether they had their webcam turned on in their last online meeting (*yes* or *no*).

The descriptive norm was measured for the last online meeting the person had participated in. It was assessed as the percentage of participants who had their webcams turned on in that specific meeting. Answers were provided on a 5-point scale with the categories 1 (*Nobody*), 2 (*Not many, ~25%*), 3 (*Half, ~50%*), 4 (*Almost all, ~75%*), and 5 (*All*).

The injunctive norm was assessed with one item (*Other participants in the online meeting expect me to turn my webcam on*) on a 5-point scale from 1 (*do not agree*) to 5 (*fully agree*).

For further analyses, we assessed whether individuals were more likely to turn on the webcam if they were explicitly instructed to do so (*I turn on my webcam when it had explicitly been recommended that I do so before*).

We assessed the number of participants in the meeting as a control variable with six options (*2 to 3, 4 to 6, 7 to 10, 11 to 20, 21 to 50, more than 50*).

Furthermore, potential reasons for not activating a webcam in an online meeting were assessed. Therefore, participants who had previously indicated that they would not always turn on their webcam in online meetings were asked to select reasons why they do not use their webcam (*I do not use my webcam, because …*). They could choose from a list of five reasons and were instructed to select all the reasons that applied to them. In addition, they had the possibility to state other reasons. The options were, for example, *doing other things on the side* or *feeling observed*.

## Results

Tables 1, 2 present descriptive statistics and intercorrelations between the relevant variables for the university and professional groups.

**Table 1:** Means, standard deviations, and correlations in the university group.

Variable	*M*	*SD*	1	2	3
1. Webcam use	0.45	0.50			
2. Descriptive norm	1.73	1.40	0.74^***^		
3. Injunctive norm	2.67	1.16	0.30^***^	0.42^***^	
4. Number of participants in meeting	2.88	1.48	−0.38^***^	−0.48^***^	−0.15^*^

* *p* < 0.05;

*** *p* < 0.001.

**Table 2:** Means, standard deviations, and correlations in the professional group.

Variable	*M*	*SD*	1	2	3
1. Webcam use	0.71	0.46			
2. Descriptive norm	2.56	1.36	0.75^***^		
3. Injunctive norm	3.21	1.11	0.56^***^	0.51^***^	
4. Number of participants in meeting	1.91	1.38	−0.21^*^	−0.29^**^	0.05

* *p* < 0.05;

** *p* < 0.01;

*** *p* < 0.001.

The mean values for webcam use and both social norms were higher in the professional group, whereas the number of participants in a particular meeting was lower in this group. In both groups, the descriptive norm was strongly correlated with webcam use in a specific meeting. The injunctive norm was also positively correlated with webcam use and the descriptive norm in both groups. The number of participants in the meeting was negatively correlated with both types of social norms and individual webcam activation in the university group, whereas in the professional group, it was correlated with individual webcam activation and the descriptive norm but not the injunctive norm.

### Results of the logistic regression analyses

To examine the relationship between descriptive as well as injunctive norms and webcam activation in a meeting, hierarchical logistic regression analyses were computed. The variance inflation factors in both groups were below 5, which indicates that the variables were not affected by multicollinearity. Table 3 presents the results of the analyses in the professional and university groups. For professionals, both the descriptive norm, *B* = 1.47, SE(*B*) = 0.30, *p* < 0.001, and the injunctive norm, *B* = 1.40, SE(*B*) = 0.43, *p* = 0.001, significantly predicted webcam activation in a meeting, whereas the group size was not related to webcam activation. The model correctly predicted webcam activation for 91.13% of all participants. In the university group, only the descriptive norm significantly predicted webcam activation, *B* = 1.78, SE(*B*) = 0.27, *p* < 0.001. The factor e^B^ indicates how the odds ratio (i.e., the probability that a person belongs to the group of participants with their webcams on) changes when the predictor increases by one point. In the university group, e^B^ = 5.92 for the descriptive norm. That is, with a 25% increase in participants with activated webcams (one point on the scale), it became 5.92 times more likely that an individual would decide to turn on their webcam, too. Again, the total number of participants in a meeting did not explain additional variance in webcam activation behavior. The model correctly predicted webcam activation for 87.18% of all participants in the university group.

We further found that when participants in a meeting were explicitly asked to turn on their webcam, 81.84% of respondents indicated they would comply with this request (“agree” or “fully agree”).

**Table 3:** Results of the logistic regression analyses for both groups.

Professionals	*B*	SE(*B*)	e^B^	95% CI for e^B^	Z	*p*
Descriptive norm	1.47	0.30	4.37	[2.55; 8.53]	4.86	<0.001
Injunctive norm	1.40	0.43	4.04	[1.84; 10.40]	3.22	0.001
Participants in meeting	−0.27	0.26	0.76	[0.45; 1.28]	−1.03	0.303
AIC						66.46
**University**						
Descriptive norm	1.78	0.27	5.92	[3.66; 10.57]	6.62	<0.001
Injunctive norm	−0.05	0.22	0.95	[0.62; 1.45]	−0.23	0.819
Participants in meeting	−0.17	0.18	0.85	[0.59; 1.21]	−0.92	0.356
AIC						142.94

Predictors are unstandardized.

### Reasons for not turning on the webcam

**Figure 1:**
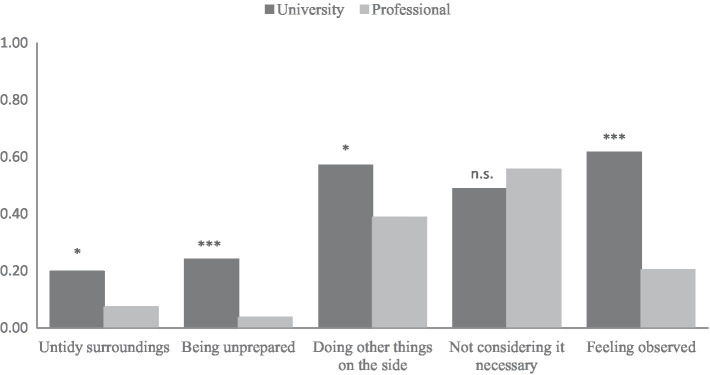
Reasons for not turning on the webcam in the university and professional groups. ^*^p < 0.05; ^***^p < 0.001.

We additionally assessed participants’ reasons for not turning on their webcams in both groups. Figure 1 illustrates the mean values of the reasons for both groups.

The most frequently reported reasons were not considering it necessary to turn on a webcam (both groups), doing other things on the side (university group), and feeling observed (university group). Except for “not considering it necessary,” the frequency of all reasons differed significantly between the two groups. In the university group, untidy surroundings were mentioned more frequently than in the professional group (*M*_Uni_ = 0.20, *SD* = 0.40; *M*_Prof_ = 0.07, *SD* = 0.26), *t*(146) = −2.44, *p* = 0.016. Furthermore, the university group more often stated that they were not prepared to turn on the webcam (*M*_Uni_ = 0.24, *SD* = 0.43; *M*_Prof_ = 0.04, *SD* = 0.19), *t*(184) = −4.88, *p* < 0.001. They also more frequently reported they would do other things on the side (*M*_Uni_ = 0.57, SD = 0.50; *M*_Prof_ = 0.39, *SD* = 0.49), *t*(99) = −2.29, *p* = 0.024. Lastly, the university group also more often reported feeling observed as a reason (*M*_Uni_ = 0.62, *SD* = 0.49; *M*_Prof_ = 0.20, *SD* = 0.41), *t*(117) = −5.93, *p* < 0.001.

In addition, 30 participants (24 from the university group, 6 from the professional group) indicated other reasons for not turning on their webcams. Most of them mentioned technical issues (*n* = 8), others referred to problems with the internet connection or the desire to save bandwidth (*n* = 6). Privacy and / or appearance were a reason to keep the camera turned off for 7 persons, whereas another 7 indicated social norms as a reason not to turn on the webcam. In addition, sustainability (*n* = 1), distraction (*n* = 2), multitasking (*n* = 1) and shyness (*n* = 1) were mentioned as reasons.

## Discussion

This study demonstrated that descriptive norms are related to the decision to turn on the webcam in an online meeting in university and professional contexts. The larger the percentage of participants who had their webcams turned on, the more likely it was that the study participant decided to activate their webcam, too. In a professional context but not in a university context, injunctive norms had an additional influence on webcam use. Finally, the reasons for why participants preferred not to turn on their webcams differed between meetings in university and professional contexts.

One explanation that drives some people more than others not to turn on their cameras might be the still prevailing discrepancies between face-to-face meetings and videoconferences. The human brain is designed to facilitate face-to-face communication (e.g., Kock, 2004). Therefore, the more a communication channel deviates from this natural form of communication, the greater the cognitive effort that is required to use it. Some other studies have also supported the fact that there are circumstances where webcam use is simply not beneficial. For instance, Wegge (2006) observed performance reductions in pupils from seeing their own image. Adults also tend to look at their own image during a large part of a meeting (Fossilien and Duffy, 2020). However, in many cases, the webcam use of most people participating in a videoconference will be beneficial, particularly for tasks that involve complex or ambiguous problems that need to be solved by the group (Daft and Lengel, 1983; Hofmann et al., 2013). For newly formed groups, switching on the webcam could make it easier to connect emotionally with each other (Bedenlier et al., 2021). Thus, making the social norm to use a webcam more salient in videoconferences can facilitate engagement and joint discussions in groups of students and professionals. It should also be noted that the immediately visible social norm can be highly skewed technically through the way the software presents the participants (i.e., if in a larger meeting, the videoconferencing tool only shows the tiles of those participants who have turned their webcams on). This opens up opportunities to motivate participants to turn on their webcams, even if the social norm would not favor this behavior in the group.

However, meeting organizers should carefully consider the circumstances in which the benefits of webcam use in a meeting outweigh the disadvantages. For instance, if the goal of a meeting is to share information, a high level of media richness is not necessary.

The injunctive norm did not explain further variance in webcam use in the university group. This could be due to the larger average number of participants in this group, in which—compared to smaller groups or work teams—participants often do not know each other. Another reason could be the speaker-audience context which is often present in university lectures in Germany.

Besides these factors, meeting organizers should also consider the environmental consequences of meetings when deciding to motivate participants to turn on their webcams or not. Although virtual meetings already drastically reduce CO_2_ emissions, turning all of the webcams off in a 60-person meeting can save another 9 kg of CO_2_ (which equals a 45 km car ride).^2^ Even though this aspect was only explicitly mentioned by one participant in this study, it could become a relevant factor influencing the decision to use a webcam in the future.

### Limitations and future research

Whereas our findings are in line with the few studies that have been conducted to date, there are several limitations to consider. The majority of participants indicated they would turn on their webcam if someone had explicitly recommended they do so before, but we did not control for whether such a recommendation had been made at their last meeting. Moreover, we used an *ad hoc* sample and tested our hypotheses with a correlative design, which limits the generalizability of our results and does not allow us drawing inferences about causality. Therefore, future studies should test whether these findings can be replicated using an experimental study and a heterogeneous sample. We did not control for the profession and the status of the participant (e.g., employee or leader) or the meeting type (e.g., whether an interaction between the participants was desired or helpful). For instance, Marlow et al. (2017) found that whereas presenters perceive audience visibility as useful, it is not equally profitable for the audience members to see each other. Therefore, future studies could elaborate on what the appropriate media mix might be for satisfactory and efficient communication. Although we offered participants the opportunity to enter additional reasons for not using a webcam in an open text field, many only selected reasons from the list presented. This list is not exhaustive, so a more thorough analysis of possible reasons should be the subject of future studies.

## Conclusion

So far, the influence of social norms on webcam activation has been underestimated. Previous studies have mostly reported privacy concerns, technical problems, and similar reasons (Bedenlier et al., 2021). Castelli and Sarvary (2021) drew attention to the influence of social norms; however, these were also stated less frequently in their study in comparison with other reasons. It can therefore be assumed that social norms effectively influence behavior unconsciously. Increasing the salience of such social norms in a meeting can motivate participants to turn on their webcams.

## Data availability statement

The raw data supporting the conclusions of this article will be made available by the authors, without undue reservation.

## Ethics statement

Ethical review and approval were not required for the study on human participants in accordance with the local legislation and institutional requirements. Written informed consent from the participants’ legal guardian/next of kin was not required to participate in this study in accordance with the national legislation and the institutional requirements.

## Author contributions

SO supervised the project. GV collected the data. SZ and GV performed the statistical analyses. SZ drafted the manuscript. All authors contributed to the study design. All authors contributed to the article and approved the submitted version.

## Funding

SZ and SO gratefully acknowledge funding provided by the Stiftung Innovation in der Hochschullehre (project number FBM2020-EA-1670-01800).

## Acknowledgments

The authors would like to thank Jane Zagorsky for her language support.

## Conflict of interest

The authors declare that the research was conducted in the absence of any commercial or financial relationships that could be construed as a potential conflict of interest.

## Publisher’s note

All claims expressed in this article are solely those of the authors and do not necessarily represent those of their affiliated organizations, or those of the publisher, the editors and the reviewers. Any product that may be evaluated in this article, or claim that may be made by its manufacturer, is not guaranteed or endorsed by the publisher.

## Footnotes

^1^Zabel, S., Schlaile, M. P., and Otto, S. (2021). Breaking the Chain with Individual Gain? Investigating the Moral Intensity of COVID-19 Digital Contact Tracing. Manuscript submitted for publication.

^2^Obringer et al. (2021) calculated CO2 emissions when using a webcam with high video quality in a 1-hr meeting. With 60 webcams, the CO2 footprint equals 9.4 kg, whereas with the webcams turned off, in the same meeting, CO2 emissions were reduced to 377 g. For a gasoline-powered car with 5 L gasoline consumption per 100 km, this savings corresponds to driving about 45 km.
